# Thrombomodulin Shapes Thrombo‐Inflammatory Interactions in Lupus Nephritis

**DOI:** 10.1111/sji.70158

**Published:** 2026-10-06

**Authors:** Mads Lamm Larsen, Lea Dich Pedersen, Annette Gudmann Hansen, Trine Korsgaard Hejlesen, Anders Bengtsson, Anne Voss, Ellen M. Hauge, Susan Due Kay, Bent Deleuran, Julie B. Larsen, Steffen Thiel, Anne Troldborg

**Affiliations:** ^1^ Department of Biomedicine Aarhus University Aarhus Denmark; ^2^ Department of Rheumatology Aarhus University Hospital Aarhus Denmark; ^3^ Department of Medicine Regional Hospital Horsens Horsens Denmark; ^4^ Department of Clinical Sciences, Rheumatology Lund University, Skåne University Hospital Lund Sweden; ^5^ Department of Rheumatology Odense University Hospital Odense Denmark; ^6^ Department of Clinical Research University of Southern Denmark Odense Denmark; ^7^ Department of Clinical Medicine Aarhus University Aarhus Denmark; ^8^ Department of Clinical Biochemistry Aarhus University Hospital Aarhus Denmark

## Abstract

Lupus nephritis (LN) confers a disproportionate thrombotic risk in systemic lupus erythematosus (SLE) that is not fully explained by conventional cardiovascular risk factors. The underlying molecular mechanisms remain largely unknown. We aimed to characterize the thrombo‐inflammatory profiles in LN compared with SLE without nephritis (SLE‐NonLN), and contextualized these findings to primary antiphospholipid syndrome (APS), primary Sjögren's disease (SjD), and healthy controls. In a cross‐sectional design, we included 325 patients with SLE from two Danish cohorts, 10 with primary APS, 45 with primary SjD, and 100 healthy controls. A predefined thrombo‐inflammatory panel encompassing endothelial and platelet activation, coagulation, fibrinolysis, and complement pathways was assessed using immunoassays. Group differences were adjusted for clinical covariates and multiple testing. Multivariate patterns were examined using principal component analysis, correlation networks, and logistic regression. Thrombomodulin levels were higher in SLE‐LN than in SLE‐NonLN and remained associated with LN after multiple testing correction and adjustment for age, treatment exposure, and disease activity. Thrombomodulin showed a modest association with albuminuria but not with disease activity, anti‐double‐stranded DNA antibody levels, or secondary APS. Correlation networks demonstrated a dense, integrated thrombo‐inflammatory structure in SLE‐LN. In multivariable logistic regression, thrombomodulin and prednisolone use emerged as independent factors associated with LN. LN is characterized by a distinct thrombo‐inflammatory profile dominated by endothelial activation, with thrombomodulin emerging as the most consistent biological marker of renal involvement in SLE. The coordinated alterations observed across multiple pathways highlight the potential value of systems‐oriented approaches for advancing mechanistic understanding in LN.

## Graphical abstract

Lupus nephritis is characterized by a distinct thrombo‐inflammatory profile dominated by endothelial activation, with thrombomodulin emerging as the most consistent biological marker of renal involvement in systemic lupus erythematosus.

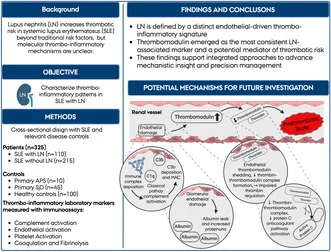

## 1 Introduction

Systemic lupus erythematosus (SLE) is a chronic multisystem autoimmune disease affecting virtually any organ, including the skin, joints, lungs, central nervous system, kidneys, and the vascular endothelium [1]. In addition to immune‐mediated tissue injury, patients with SLE have a markedly increased risk of arterial and venous thrombosis, which represents a major contributor to long‐term morbidity and mortality [2, 3].

Antiphospholipid syndrome (APS) is a well‐established driver of thrombotic risk in SLE and is present in approximately 20% of patients as a secondary condition [4]. APS is defined by persistent antiphospholipid antibodies (aPL) in combination with thrombotic events or pregnancy morbidity [5]. Although APS accounts for a substantial proportion of thrombotic events in SLE, a considerable burden of thrombosis occurs in patients without APS, indicating that additional disease‐specific mechanisms contribute to vascular risk [6].

Primary Sjögren's disease (SjD) shares several immunopathogenic features with SLE and APS, including B‐cell hyperactivity, complement system involvement, and autoantibody production [7, 8]. However, SjD is generally associated with a lower thrombotic risk and a lower prevalence of aPL [9, 10]. This makes SjD a useful disease comparator when investigating mechanisms that may specifically predispose patients with SLE, and particularly its subgroups, to thrombo‐inflammatory complications.

Lupus nephritis (LN), affecting roughly 40% of patients with SLE, remains one of its most severe manifestations and is associated with substantial comorbidity, chronic kidney disease, higher glucocorticoid exposure, and reduced survival [11]. Importantly, LN represents an independent risk factor for thrombosis in SLE, even after accounting for aPL and traditional cardiovascular risk factors [12, 13, 14]. Although impaired renal function is known to increase the thrombotic risk in the general population [15, 16], it does not fully explain the disproportionately high incidence of thromboembolic events observed in patients with LN, including events occurring in the absence of aPL [17, 18]. These observations suggest that LN may harbor additional, disease‐specific thrombo‐inflammatory mechanisms beyond those attributable to renal dysfunction and aPL alone.

Several individual pathways implicated in thrombosis and inflammation, including complement activation, endothelial dysfunction, platelet activation, coagulation, and fibrinolysis, have been described in SLE and LN [19, 20]. However, most studies have examined these systems independently, limiting insight into their interactions at the systemic level. Whether LN is characterized by a distinct or more tightly interconnected pattern of thrombo‐inflammatory pathway activation compared with SLE without renal involvement (SLE‐NonLN) remains largely unknown. Furthermore, it is unclear how such patterns compare with those observed in related autoimmune diseases such as APS and SjD.

In this study, we investigated thrombo‐inflammatory profiles across a large, well‐characterized SLE cohort. We included patients with primary APS and primary SjD, as well as healthy controls, to provide descriptive biological context for the findings observed within SLE. Using a predefined laboratory panel capturing endothelial and platelet activation, coagulation, fibrinolysis, and complement pathway activity, we aimed to identify biological features that distinguish SLE‐LN from SLE‐NonLN and to assess whether isolated pathway perturbations or enhanced cross‐pathway interactions characterize LN. By integrating univariate, multivariate, and network‐based analytical approaches, we sought to provide a comprehensive characterization of thrombo‐inflammatory mechanisms associated with renal involvement in SLE. We hypothesized that LN is characterized by a distinct thrombo‐inflammatory pattern compared to SLE‐NonLN.

## 2 Methods

### 2.1 Study Populations

#### 2.1.1 SLE Cohort

The SLE cross‐sectional cohort comprised prevalent patients recruited from two tertiary referral centers in Denmark: Aarhus University Hospital (*n* = 203) and Odense University Hospital (*n* = 122).

Patients in the Aarhus cohort were consecutively enrolled between 2019 and 2021 at the Department of Rheumatology, Aarhus University Hospital. Inclusion criteria were age ≥ 18 years and fulfilment of the 1997 revised American College of Rheumatology (ACR) classification criteria for SLE [21]. Exclusion criteria included inability to provide informed consent, active infection, and ongoing treatment for malignancy or infectious diseases. Blood samples, standard laboratory analyses, and clinical data including current prednisolone, hydroxychloroquine, and antithrombotic treatments were collected at inclusion.

Patients with SLE prevalent at the outpatient clinic of Odense University Hospital, Denmark (*SLE Odense Cohort*), were included in a research biobank between 2010 and 2011 after informed consent, as previously described [22].

LN was biopsy‐proven and classified according to the 2003 International Society of Nephrology/Renal Pathology Society criteria [23], unless biopsy was contraindicated or precluded due to urgent treatment. In such cases, LN was defined by significant proteinuria (> 0.5 g/day or equivalent).

Disease activity was assessed using the Systemic Lupus Erythematosus Disease Activity Index (SLEDAI), and cumulative organ damage using the SLICC/ACR Damage Index [24, 25].

#### 2.1.2 Disease Comparator Groups and Healthy Controls

Patients prevalent with primary APS (*n* = 11) and primary SjD (*n* = 45) were consecutively recruited between 2023 and 2024 at Aarhus University Hospital. Patients with APS fulfilled the 2023 ACR/EULAR classification criteria [5], and SjD patients fulfilled the 2016 ACR/EULAR criteria [8]. Participants with clinical suspicion of overlapping autoimmune disease were excluded. Exclusion criteria included pregnancy, ongoing treatment for cancer, and inability to provide informed consent. Healthy controls (*n* = 100) were recruited from the Blood Bank at Aarhus University Hospital, Denmark, in 2023. All participants were ≥ 18 years of age and provided written informed consent.

### 2.2 Blood Sampling and Laboratory Investigations

Blood samples from all participants were collected at inclusion into EDTA or citrated tubes and processed according to standardized protocols. Plasma was separated by centrifugation, aliquoted, and stored at −80°C until analysis. For the SLE Odense cohort, only EDTA‐plasma samples were available.

A predefined thrombo‐inflammatory laboratory panel was selected based on recent systematic work from our group [19], and designed to capture key mechanisms related to platelet activation, endothelial activation, coagulation, fibrinolysis, and complement activation pathways. Lectin‐pathway complement proteins were additionally measured to assess pathway‐specific activation.

All research laboratory analyses were performed between July 2024 and January 2026 at the Department of Clinical Biochemistry, Aarhus University Hospital and the Department of Biomedicine, Aarhus University. Samples were analysed in randomized batches to minimize any bias that could be introduced by inter‐assay variability. Laboratory personnel were blinded to clinical group assignment. All measurements were performed in duplicate according to the manufacturer's instructions or established in‐house validated protocols.

#### 2.2.1 Assessment of Platelet and Endothelial Activation

Platelet activation was assessed by plasma P‐selectin (ELISA, EDTA plasma, R&D Systems, #DPSE00).

Endothelial activation was assessed using ELISA for syndecan‐1 (CD138; EDTA plasma; Nordic Biosite, #950.640.192) and thrombomodulin (CD141; EDTA plasma; Nordic Biosite, #850.720.192).

#### 2.2.2 Assessment of Coagulation and Fibrinolysis

Markers of coagulation and fibrinolysis included prothrombin fragment 1 + 2 (F1 + 2; citrated plasma; Siemens Healthcare Diagnostics, #OPBD035) and plasminogen activator inhibitor‐1 (PAI‐1; citrated plasma, Technoclone, #TC12075).

#### 2.2.3 Complement Analysis

Complement activation products and lectin‐pathway proteins were measured in EDTA plasma using in‐house time‐resolved immunofluorometric assays (TRIFMAs), except for L‐ficolin, MASP‐1/C1‐INH complexes, and C1s/C1‐INH complexes, which were quantified using commercial ELISAs (Hycult Biotech, #HK336‐02, #HK3001 and #HK399, respectively).

TRIFMA targets included C3dg, MASP‐1, MASP‐2, MASP‐3, mannan‐binding lectin (MBL), H‐ficolin, M‐ficolin, collectin‐liver‐1 (CL‐L1), collectin‐kidney‐1 (CL‐K1), and inter‐α‐trypsin inhibitor heavy chain 4 (ITIH4). Detailed assay descriptions have been published previously [26, 27, 28, 29, 30, 31, 32, 33, 34, 35, 36].

Sample dilution and plate loading were automated using a JANUS pipetting robot (PerkinElmer). Measurements with a coefficient of variation (CV) > 15% were repeated. Inter‐assay CVs based on internal controls were consistently < 15%.

#### 2.2.4 Routine Laboratory Data

Routine clinical and biochemical parameters were obtained at inclusion, including anti–double‐stranded DNA antibodies (anti‐dsDNA), C‐reactive protein, creatinine, lactate dehydrogenase, alanine transaminase, hematologic indices, immunoglobulin G, complement C3 and C4, and urine albumin measurements.

### 2.3 Statistics

All analyses were performed in R (v4.5.1). Continuous variables are presented as mean ± standard deviation (SD) or median with interquartile range (IQR), as appropriate. Categorical variables are presented as counts and percentages.

Potential differences between SLE cohorts related to the inclusion period, sample availability, and clinical characteristics were assessed and addressed in sensitivity analyses.

Group comparisons between SLE‐LN and SLE‐NonLN were performed using the Mann–Whitney *U* test or 2‐sample *t*‐test on raw or log‐transformed data, depending on distribution. Multiple testing across laboratory variables was addressed using false discovery rate (FDR) and Bonferroni correction.

For principal component analysis (PCA), laboratory variables were restricted to complete cases, log‐transformed, centered, and scaled. The first two principal components were visualized.

Spearman's rank correlation coefficients were calculated using pairwise complete observations within each disease group. Correlation networks were constructed using statistically significant associations (*p* ≤ 0.05) and visualized using tidygraph and ggraph. Sensitivity analysis was performed using FDR‐adjusted *p*‐values, applied separately within each disease group.

Multivariable linear regression was used to assess adjusted associations between disease group and individual laboratory markers, with inclusion of relevant clinical covariates (age, disease activity, and treatment exposures). Model assumptions were evaluated using residual diagnostics.

Multivariable logistic regression was used to identify associations with LN within the SLE cohort. All thrombo‐inflammatory variables and selected clinical covariates were included in an initial complete model. Model reduction was performed using Akaike Information Criterion (AIC)‐based stepwise selection, and sensitivity analyses were conducted using Least Absolute Shrinkage and Selection Operator (LASSO) penalized logistic regression. Multicollinearity, logit linearity, and influential observations were assessed. Discriminatory performance was evaluated using receiver operating characteristic (ROC) curves. The disease comparator groups and healthy controls were included primarily to provide descriptive context for the findings, and formal statistical comparisons across all disease groups were not performed.

Only complete‐case analyses were applied. All statistical tests were 2‐sided, and *p*‐values < 0.05 were considered statistically significant.

### 2.4 Ethical Statement

The study was performed in accordance with the Declaration of Helsinki II. All participants provided written informed consent. Ethical approval was granted by the Regional Committee on Health Research Ethics for the Central Denmark Region (case no. 1–10–72‐188‐22 and no. 1–10–72‐288‐18) and the Regional Committees on Health Research Ethics for Southern Denmark (case no. S‐2010 0015). Data handling complied with the General Data Protection Regulation and the Danish Data Protection Act.

## 3 Results

### 3.1 Patient and Control Characteristics

Baseline demographic, clinical, and laboratory characteristics of patients and controls are summarized in Table 1. The SLE cohort (*n* = 325) had a median age of 47 years, was predominantly female (90%), and consisted mainly of individuals of White ethnicity (94%). Median disease duration was 10 years, and LN was present in 110 patients (34%). Kidney biopsy data were available for 48 patients overall, including 47 patients classified as SLE‐LN.

**TABLE 1:** Demographics and Characteristics.

	Variable	SLE (ALL) *N* = 325	SLE (LN) *N* = 110	SLE (Non‐LN) *N* = 215	APS *N* = 10	SjD *N* = 45	HC *N* = 100
General demographics	Age	47 (37, 58)^a^	42 (35, 56)^a^	49 (38, 60)^a^	38 (35, 54)^a^	59 (45, 69)^a^	38 (28, 52)^a^
	Female sex	293 (90%)	96 (87%)	197 (92%)	7 (70%)	37 (82%)	67 (67%)
	Ethnicity, white	306 (94%)	100 (91%)	206 (96%)	10 (100%)	45 (100%)	NA
	Years of Disease	10 (4, 19)	13 (6, 20)	9 (3, 19)	NA	7 (3, 11)	
SLE characteristics	Total ACR score	6 (5, 7)	7 (6, 8)	6 (5, 7)			
	ACR 1 (Malar Rash)	205 (63%)	76 (69%)	129 (60%)			
	ACR 2 (Discoid Lupus)	24 (7.4%)	4 (3.6%)	20 (9.3%)			
	ACR 3 (Photosensitivity)	195 (60%)	62 (56%)	133 (62%)			
	ACR 4 (Ulcers)	120 (37%)	39 (35%)	81 (38%)			
	ACR 5 (Arthritis)	295 (91%)	101 (92%)	194 (90%)			
	ACR 6 (Serositis)	125 (38%)	50 (45%)	75 (35%)			
	ACR 7 (Nephritis)	110 (34%)	110 (100%)	0 (0%)			
	Kidney biopsy available	48 (15%)^d^	47 (43%)^d^	1 (0.5%)^d^			
	WHO Class I‐II		6 (13%)	0			
	WHO Class III, IV		31 (65%)	0			
	WHO Class V		5 (11%)	0			
	Other^b^		5 (11%)	1 (100%)			
	ACR 8 (CNS)	39 (12%)	16 (15%)	23 (11%)			
	ACR 9 (Haematological)	245 (75%)	91 (83%)	154 (72%)			
	ACR 10 (Immunological)	297 (91%)	105 (95%)	192 (89%)			
	ACR 11 (ANA)	324 (100%)	110 (100%)	214 (100%)			
	SLEDAI score	4 (2, 4)	2.5 (1, 6)	2 (1, 4)			
	Physician global assessment	0 (0, 2)^d^	0 (0, 1)^d^	0 (0, 1)^d^			
	SLICC damage score	1 (0, 2)	1 (0, 2)	1 (0, 2)			
	Secondary APS	71 (22%)	26 (24%)	45 (21%)			
	Secondary SjD	41 (20%)^d^	7 (12%)^d^	34 (24%)^d^			
APS characteristics	Thrombotic APS				6 (60%)		
	Obstetric APS				4 (40%)		
	EULAR/ACR 2023 class. score				9 (8, 15)		
Sjögren characteristics	EULAR/ACR 2016 class. score					5 (5, 6)	
	ESSDAI score					2 (1, 8)	
Treatments at inclusion	Prednisolone	158 (50%)	70 (64%)	88 (42%)	0 (0%)	5 (11%)	
	Hydroxychloroquine	213 (72%)	71 (75%)	142 (70%)	0 (0%)	17 (38%)	
	Antithrombotic Treatment	85 (42%)^d^	28 (47%)^d^	57 (40%)^d^	6 (60%)	16 (36%)	
	Antiplatelet therapy	NA	NA	NA	1 (10%)	10 (22%)	
	Anticoagulant therapy^c^	NA	NA	NA	6 (60%)	5 (11%)	
	Lipid‐lowering therapy	38 (19%)^d^	10 (17%)^d^	28 (20%)^d^	NA	NA	
	Anti‐hypertensive therapy	60 (30%)^d^	22 (37%)^d^	38 (27%)^d^	NA	NA	
Serology	ANA positive	324 (100%)	110 (100%)	214 (100%)	2 (33%)	40 (93%)	
	Lupus anticoagulant positive	56 (32%)^d^	17 (34%)^d^	39 (31%)^d^	10 (100%)	3 (7%)	
Laboratory values	Anti‐dsDNA titre (IU/mL)	9 (2, 33)^d^	16 (8, 60)^d^	7 (1, 25)^d^	NA	NA	
	Lactate dehydrogenase (U/L)	189 (164, 219)^d^	194 (164, 228)^d^	187 (165, 212)^d^	NA	190 (156, 209)	
	Alanine aminotransferase (U/L)	21 (16, 28)^d^	22 (17, 29)^d^	21 (16, 28)^d^	NA	22 (18, 26)	
	creatinine (μmol/L)	62 (55, 72)^d^	70 (58, 80)^d^	61 (55, 70)^d^	NA	66 (59, 79)	
	C‐reactive protein (mg/L)	4.0 (4.0, 4.2)^d^	4.0 (2.1, 4.1)^d^	4.0 (4.0, 4.7)^d^	NA	1.1 (1.1, 4.0)	
	C3 (g/L)	0.89 (0.73, 1.07)	0.85 (0.69, 1.02)	0.90 (0.76, 1.10)	NA	1.10 (1.00, 1.20)	
	C4 (g/L)	0.12 (0.07, 0.20)	0.12 (0.07, 0.21)	0.13 (0.08, 0.19)	NA	0.18 (0.14, 0.22)	
	Immunoglobulin G (g/L)	11.5 (9.4, 14.0)^d^	11.2 (8.5, 13.8)^d^	11.7 (9.7, 14.0)^d^	NA	13.2 (10.2, 17.7)	
	Leukocyte count (×10^9^/L)	5.50 (4.40, 7.25)^d^	6.00 (4.98, 7.81)^d^	5.35 (4.29, 7.10)^d^	NA	5.20 (4.30, 6.50)	
	Lymphocyte count (×10^9^/L)	1.27 (0.91, 1.74)^d^	1.13 (0.75, 1.86)^d^	1.28 (0.95, 1.73)^d^	NA	1.33 (1.20, 1.69)	
	Haemoglobin (mmol/L)	8.40 (7.70, 8.80)^d^	8.40 (7.65, 8.80)^d^	8.40 (7.80, 8.70)^d^	NA	8.10 (7.60, 8.80)	
	Platelet count (×10^9^/L)	237 (203, 280)^d^	248 (207, 283)^d^	233 (203, 279)^d^	NA	241 (208, 275)	
	Spot‐urine ACR (mg/g)	5 (2, 20)^d^	74 (10, 378)^d^	5 (3, 7)^d^	NA	NA	
	Urine ACR > 500 mg/g (spot/day)	23 (7.2%)	22 (20%)	1 (0.5%)	NA	NA	

Abbreviations: ACR, American College of Rheumatology; ANA, antinuclear antibody; APS, aniphoshoplipd syndrome (without SLE); dsDNA, double‐stranded DNA antibody; ESSDAI, EULAR Sjögren's Syndrome Disease Activity Index; EULAR, European Alliance of Associations for Rheumatology; SjD, primary Sjögren's disease; SLE, systemic lupus erythematosus; SLEDAI, Systemic Lupus Erythematosus Disease Activity Index; SLICC, Systemic Lupus International Collaborating Clinics Damage Index.

^a^ Median (Q1, Q3); *n* (%).

^b^ Thrombotic microangiopathy, segmental necrosis, or mesangial glomerulonephritis.

^c^ Vitamin K antagonist or direct oral anticoagulant treatment.

^d^ Only available data from the Aarhus cohort (*n* = 203).

Twenty‐two percent of patients had secondary APS. Overall, disease activity was low, with a median SLEDAI score of 4. At inclusion, 50% of patients received prednisolone, and 72% received hydroxychloroquine. Among patients with available data, 42% received antithrombotic therapy.

Comparisons between recruitment sites are presented in Table S1. Although the Aarhus and Odense cohorts differed in selected clinical manifestations, disease activity, and treatment exposure, key demographic variables were similar. MASP‐3 was the only laboratory variable that differed between sites without an apparent clinical explanation. Sensitivity analyses excluding MASP‐3 data from the Odense cohort did not materially affect group comparisons, correlation analyses, or regression models. We therefore retained the Odense MASP‐3 measurements in the primary analyses. Comparison between SLE‐LN patients with (*n* = 47) and without (*n* = 63) available kidney biopsy data is presented in Table S2. Patients with available biopsy data were younger, had a higher SLEDAI score, a lower SLICC score, and less secondary APS. These differences primarily reflect that biopsy data were only available from the Aarhus cohort.

Regarding disease comparator groups and controls, one primary APS patient was excluded due to a diagnosis of SLE within 3 months after inclusion, yielding a final APS study population of 10. Descriptively, compared with the SLE cohort, both patients with APS and healthy controls were younger, whereas patients with SjD were older (Table 1).

### 3.2 Thrombomodulin Levels Are Increased in Lupus Nephritis

Measurement of the thrombo‐inflammatory laboratory panel across SLE‐LN, SLE‐NonLN, APS, SjD, and healthy controls is shown in Figure 1a–d. In unadjusted analyses, syndecan‐1 and thrombomodulin were both higher in SLE‐LN compared with SLE‐NonLN (Table 2). After both FDR correction and in multivariable linear regression, adjusting for age, disease activity, prednisolone use, and antithrombotic therapy, only thrombomodulin remained associated with SLE‐LN, corresponding to a 0.26‐unit increase in log‐transformed values (95% CI: 0.08–0.44, *p* = 0.004), equivalent to approximately 29% higher circulating levels (Figure 1a).

**FIGURE 1:**
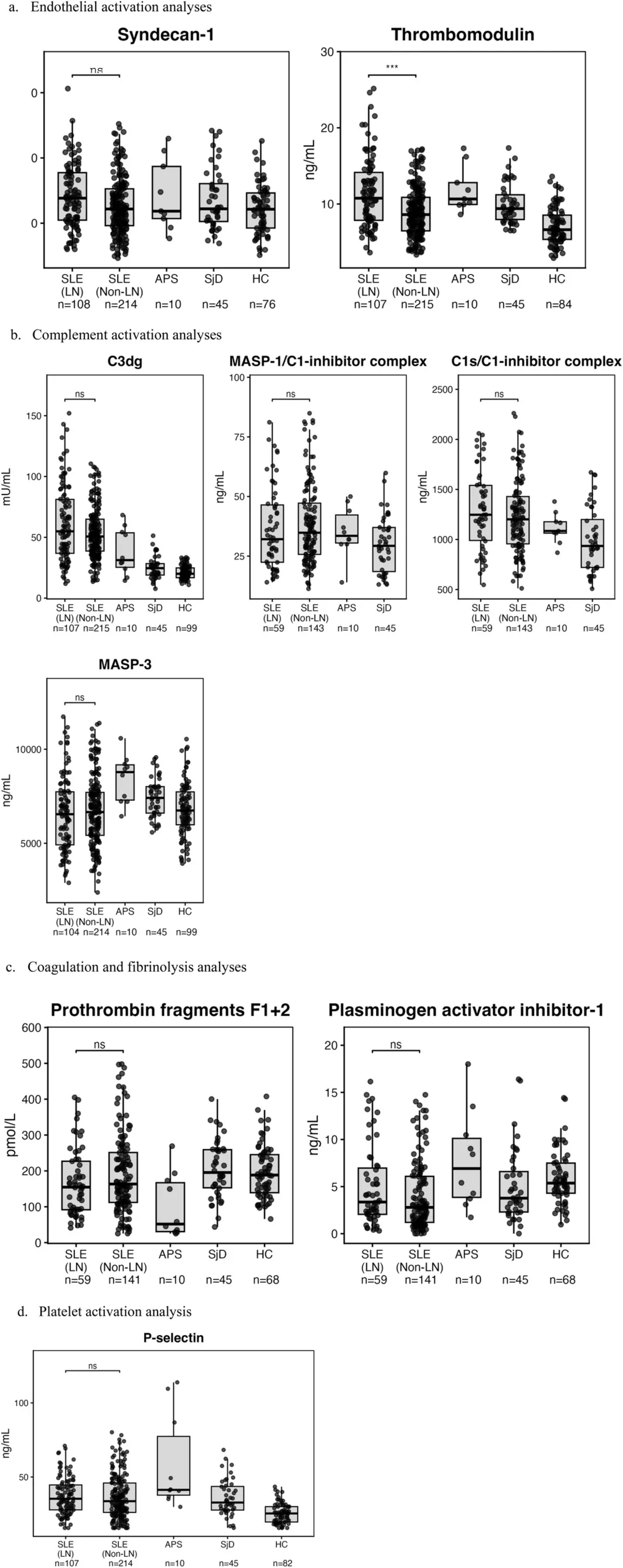
Thrombo‐inflammatory laboratory panel levels across systemic lupus erythematosus (SLE) with lupus nephritis (SLE‐LN), SLE without nephritis (SLE‐NonLN), primary antiphospholipid syndrome (APS), primary Sjögren's disease (SjD), and healthy controls (HC). Figure 1 shows levels of markers reflecting (a), endothelial activation analyses; (b), complement activation analyses; (c), coagulation and fibrinolysis analyses; and (d), platelet activation analysis. Boxplots display medians and interquartile ranges (IQR); whiskers represent 1.5 × IQR limits. Outliers beyond whiskers were removed only for visualization. Individual observations are plotted as jittered points. Statistical testing was restricted to the comparison between SLE‐LN and SLE‐NonLN using the Mann–Whitney *U* test after false discovery rate (FDR) correction; significance is shown as ns (not significant), *, **, or *** for *p* < 0.05, *p* < 0.01, and *p* < 0.001, respectively.

**TABLE 2:** Multiple‐testing‐corrected Mann–Whitney *U* test comparisons of laboratory variables between SLE lupus nephritis (SLE‐LN) and SLE without nephritis (SLE‐NonLN).

Variable	Raw *p* (Mann–Whitney LN vs. non‐LN)	FDR‐corrected *p*	Bonferroni‐corrected *p*
Thrombomodulin (CD141)	< 0.0001	< 0.0001	< 0.0001
Mannan‐binding lectin	0.0137	0.0998	0.2596
Syndecan‐1 (CD138)	0.0158	0.0998	0.2993
MASP‐1	0.0588	0.2751	1.0000
Inter‐α‐trypsin inhibitor heavy chain 4	0.0724	0.2751	1.0000
Prothrombin fragments F1 + 2	0.1158	0.3668	1.0000
MASP‐3	0.1911	0.4658	1.0000
M‐ficolin	0.2038	0.4658	1.0000
C1s/C1‐inhibitor complex	0.2485	0.4658	1.0000
Plasminogen Activator Inhibitor‐1	0.2662	0.4658	1.0000
MASP‐1/C1‐inhibitor complex	0.2697	0.4658	1.0000
C3dg	0.3228	0.5111	1.0000
MAp44	0.4814	0.5983	1.0000
Collectin‐Liver 1	0.5322	0.5983	1.0000
H‐ficolin	0.5330	0.5983	1.0000
L‐ficolin	0.5494	0.5983	1.0000
Collectin‐Kidney 1	0.5590	0.5983	1.0000
MASP‐2	0.5668	0.5983	1.0000
P‐selectin	0.8708	0.8708	1.0000

Thrombomodulin levels were not associated with SLEDAI score (Spearman's *ρ* = 0.09, *p* = 0.09) and did not differ by secondary APS status or available kidney biopsy data (*p* = 0.19 and *p* = 0.41, respectively; Mann–Whitney *U* test). A modest positive correlation was observed between thrombomodulin and UACR among patients with LN (*ρ* = 0.29, *p* = 0.029; Figure S2d).

### 3.3 Platelet, Complement, Coagulation, and Fibrinolytic Markers Across Disease Groups

No consistent differences were observed across platelet, complement, coagulation, and fibrinolytic markers between SLE‐LN and SLE‐NonLN. C3dg and C1s/C1‐INH complexes showed non‐significant tendencies toward higher levels in SLE‐LN (Figure 1b).

F1 + 2 and PAI‐1 displayed heterogeneous patterns across disease groups (Figure 1c). Neither F1 + 2 nor PAI‐1 differed significantly between SLE‐LN and SLE‐NonLN. APS patients showed lower F1 + 2 levels and higher PAI‐1 levels than other groups. In multivariable models including age, disease group, SLEDAI, and antithrombotic therapy, antithrombotic treatment was independently associated with lower F1 + 2 levels (βlog = −0.86, *p* < 0.001), corresponding to a 58% reduction. No corresponding association was observed for PAI‐1.

Plasma P‐selectin levels were descriptively increased across all autoimmune disease groups compared with healthy controls, with the highest levels observed in APS (Figure 1d).

### 3.4 Interconnected Thrombo‐Inflammatory Interaction Patterns in Lupus Nephritis

PCA, including patients with complete data (*n* = 251), demonstrated modest separation between the SLE and disease control groups, with PC1 and PC2 explaining 21.1% and 14.7% of the variance, respectively, and substantial overlap across all groups (Figure 2).

**FIGURE 2:**
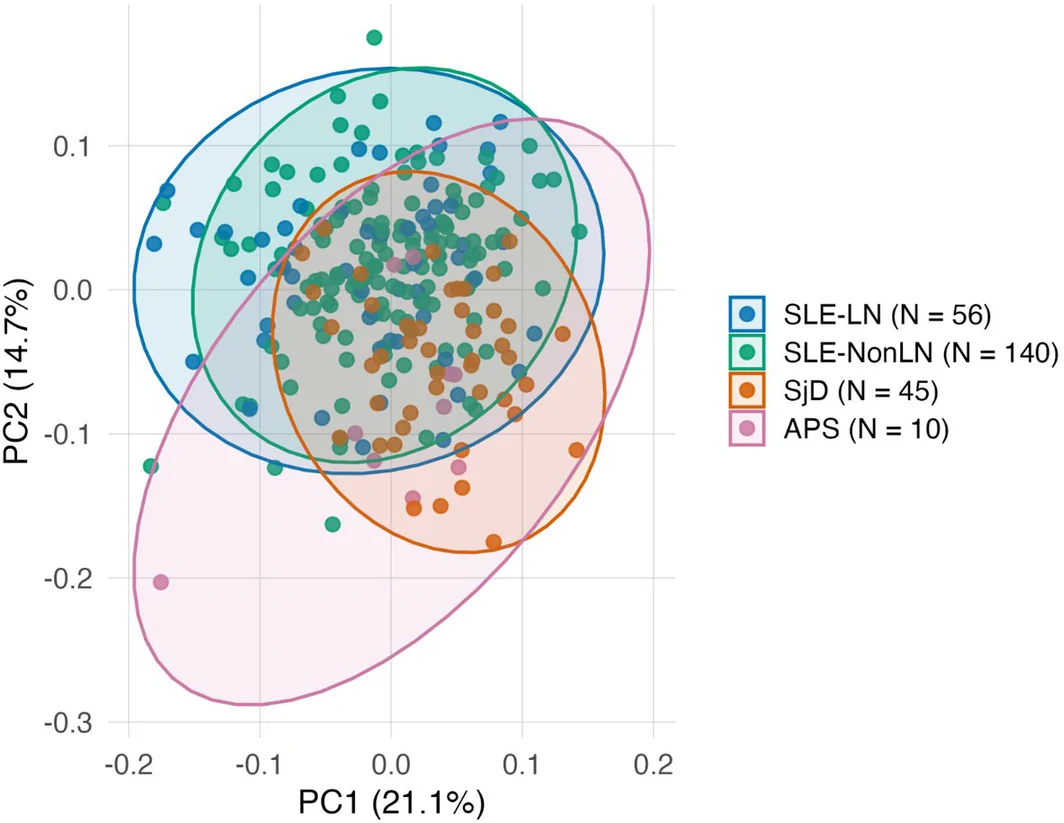
Principal component analysis (PCA) of the thrombo‐inflammatory panel in systemic lupus erythematosus (SLE) with nephritis (SLE‐LN), SLE without nephritis (SLE‐NonLN), primary antiphospholipid syndrome (APS), and primary Sjögren's disease (SjD). Points represent individual participants; ellipses show confidence regions. Only patients with complete data were included (*n* = 251). The PCA demonstrates modest group separation with notable overlap, consistent with shared clinical and inflammatory characteristics.

FDR‐adjusted Spearman correlation matrices and network visualizations are presented in Figure 3a–d. The SLE cohort displayed small networks with five significant correlations in SLE‐LN and seven in SLE‐NonLN. No significant correlations remained significant after FDR correction in APS and SjD. In unadjusted analyses using nominal *p*‐values (Figure S1), 12 significant correlations were observed in SLE‐LN, 10 in SLE‐NonLN, three in APS, and seven in SjD.

**FIGURE 3:**
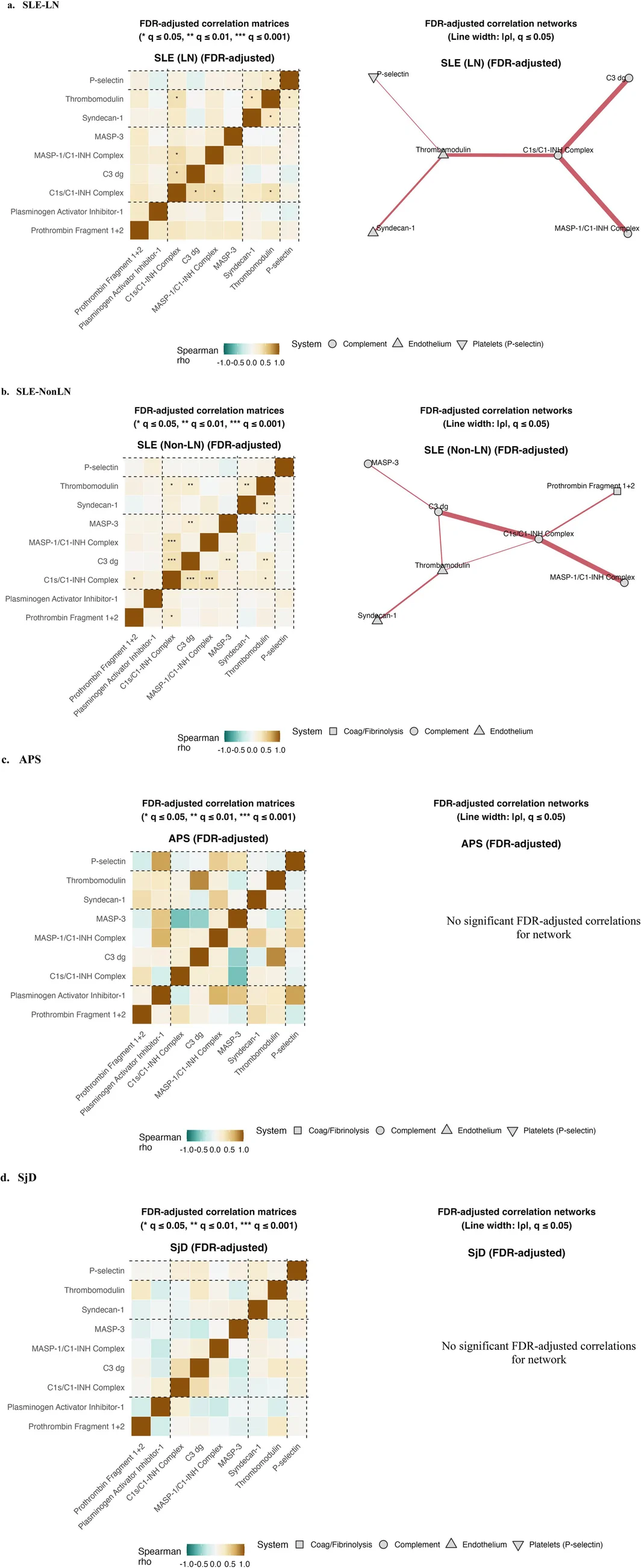
False discovery rate (FDR)‐adjusted Spearman correlation matrices and correlation networks across systemic lupus erythematosus with lupus nephritis (SLE‐LN, a), SLE without nephritis (SLE‐nonLN, b), primary antiphospholipid syndrome (APS, c), and primary Sjögren's disease (SjD, d). Heatmaps show Spearman correlation coefficients (ρ), with FDR‐adjusted significance indicated as *, **, *** for *p* < 0.05, *p* < 0.01, and *p* < 0.001, respectively. Networks display only significant correlations (FDR‐corrected *p* ≤ 0.05), with edge width proportional to |ρ| and red/blue edges denoting positive/negative associations. Biomarker nodes are grouped by biological subsystem.

### 3.5 Variables Associated With Lupus Nephritis in Multivariable Models

Multivariable logistic regression, including thrombo‐inflammatory markers and clinical covariates, identified thrombomodulin and anti‐dsDNA as independently associated with LN (Figure 4a; Table S3). Sex was tested as a covariate but was excluded from the model because it was not significantly associated with the outcome and increased estimate uncertainty due to the limited male sample size.

**FIGURE 4:**
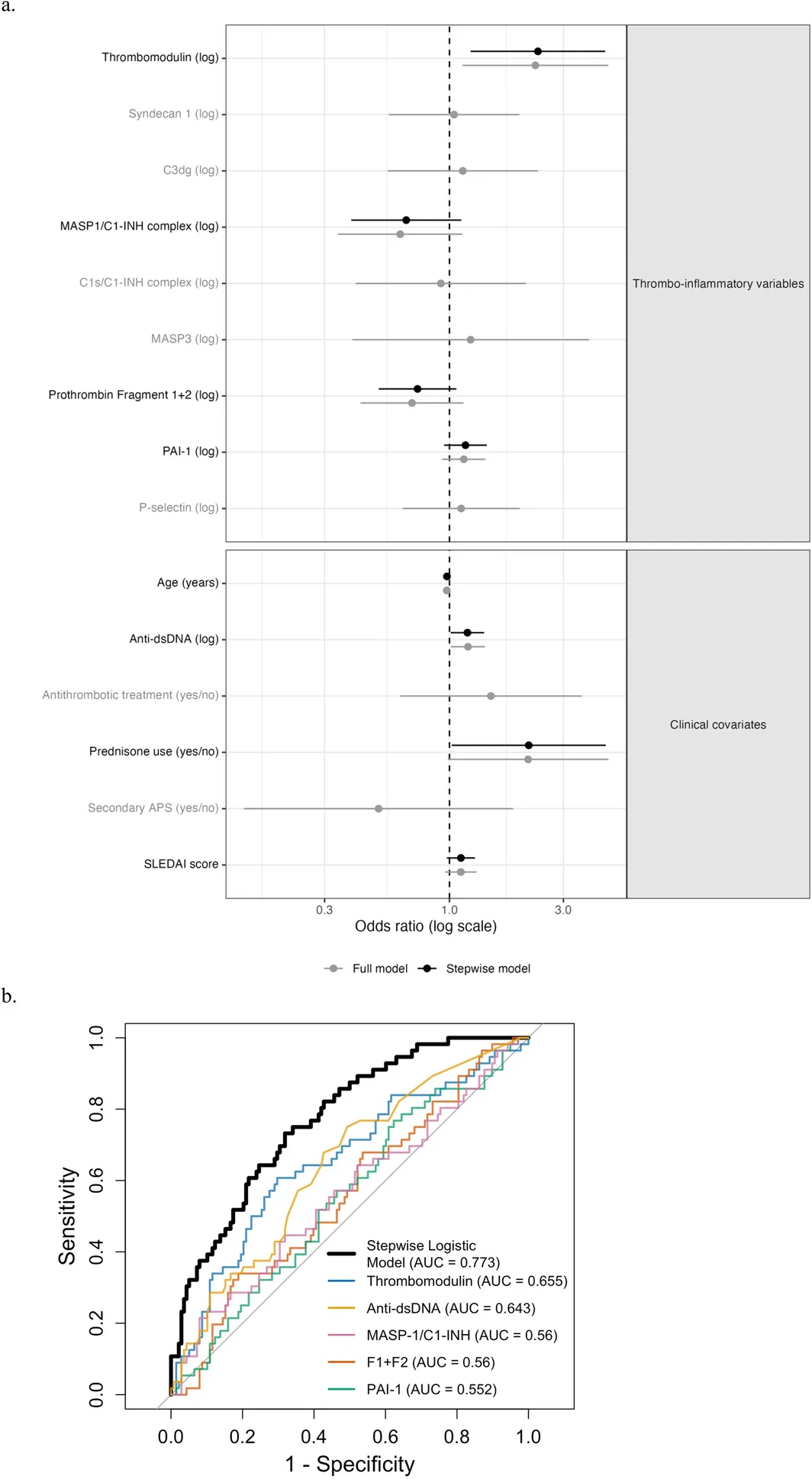
Logistic regression analysis. (a), Forest plot comparing odds ratios from the complete multivariable logistic regression model and the stepwise model for associations with lupus nephritis (LN) in systemic lupus erythematosus (SLE). Variable names and estimates shown in bold black indicate variables retained in the final stepwise model. Odds ratios (ORs) and 95% confidence intervals (CIs) are displayed on a logarithmic scale. (b) Receiver Operating Characteristic (ROC) curves of the discriminatory ability of the stepwise logistic model compared with individual thrombo‐inflammatory variables and anti–double‐stranded DNA antibody value (anti‐dsDNA). The ROC curves visualize descriptive performance and are not intended for clinical prediction. C1‐inhibitor (C1‐INH), plasminogen‐activator inhibitor (PAI)‐1, SLE disease activity index (SLEDAI).

A reduced model derived using AIC‐based stepwise selection retained thrombomodulin, MASP‐1/C1‐INH complexes, F1 + 2, PAI‐1, anti‐dsDNA, age, SLEDAI, and prednisolone use (Figure 4a, shown in bold). In this model, thrombomodulin, anti‐dsDNA, and prednisolone use were significantly associated with LN, although prednisolone use reached significance only after variable selection. ROC analysis demonstrated moderate discriminatory performance of the stepwise model (AUC = 0.773; Figure 4b). Sensitivity analyses using a LASSO‐penalized logistic regression with the same candidate variables produced a similar selection pattern: the same variables were retained as in the stepwise model, with the addition of antithrombotic treatment, which had a minimal effect size (OR = 1.06; Table S3). The AUC was comparable between the two models (0.773 vs. 0.771). These analyses were performed for descriptive purposes and were not intended for clinical prediction.

### 3.6 Assessment of Lectin Pathway Complement Activation in Lupus Nephritis

Lectin pathway proteins were examined to assess pathway‐specific complement activation in SLE‐LN. MASP‐1/C1‐INH complexes tended to be lower in SLE‐LN compared with SLE‐NonLN, although this difference did not reach statistical significance. Boxplot analyses demonstrated largely overlapping distributions of lectin pathway proteins between SLE‐LN and SLE‐NonLN, except for MBL, which was higher in SLE‐LN (Figure 5).

**FIGURE 5:**
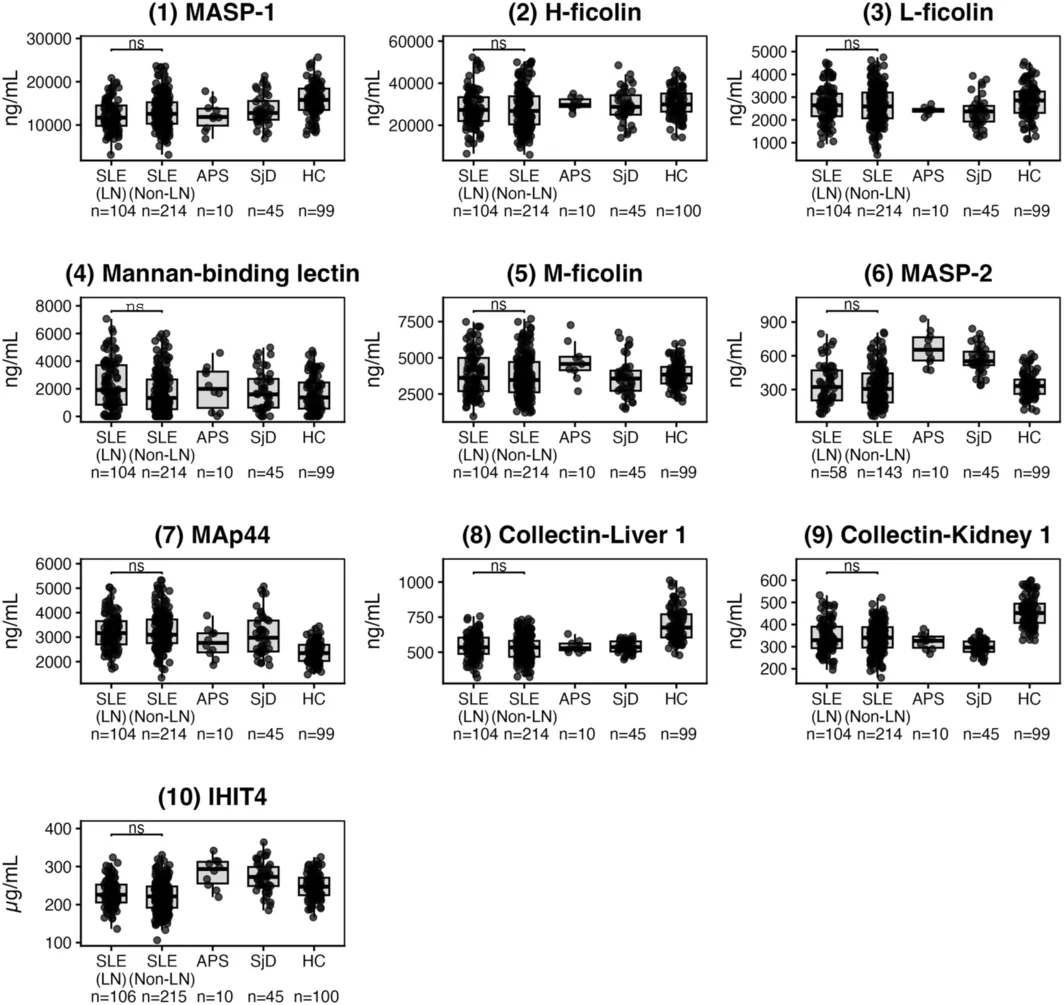
Lectin pathway complement analyses levels across systemic lupus erythematosus (SLE) with lupus nephritis (SLE‐LN), SLE without nephritis (SLE‐NonLN), primary antiphospholipid syndrome (APS), primary Sjögren's disease (SjD), and healthy controls (HC). Boxplots display medians and interquartile ranges (IQR); whiskers represent 1.5 × IQR limits. Outliers beyond whiskers were removed only for visualization. Individual observations are plotted as jittered points. Statistical testing was restricted to the comparison between SLE‐LN and SLE‐NonLN using the Mann–Whitney *U* test; significance is shown as ns (not significant), *, **, or *** for *p* < 0.05, *p* < 0.01, and *p* < 0.001, respectively. IHIT4, Inter‐α‐trypsin inhibitor heavy chain 4; MASP, Mannose‐Binding Lectin (MBL)‐Associated Serine Protease; MAp, MBL‐Associated Protein.

After correction for multiple testing using both false discovery rate and Bonferroni procedures, thrombomodulin remained the only laboratory marker that differed significantly between SLE‐LN and SLE‐NonLN (Table 2; *p* < 0.0001).

### 3.7 Associations Between Kidney Biopsy Class, Proteinuria, Anti‐dsDNA Levels, and the Thrombo‐Inflammatory Panel

To explore whether thrombo‐inflammatory profiles differed according to renal histopathology or degree of proteinuria, PCA was performed in SLE‐LN patients with available biopsy class data (WHO Class I‐II: *n* = 3; Class III‐IV: *n* = 30; Class V: *n* = 5; Figure S2a) and in patients stratified by UACR above or below 500 mg/g (Figure S2b). These analyses revealed no clear separation between biopsy classes or proteinuria categories, although patients with WHO Class V and I‐II showed some tendency toward partial clustering, suggesting subtle differences in variance structure.

Consistent with the PCA findings, boxplot analyses across WHO classes demonstrated no statistically significant between‐group differences for individual thrombo‐inflammatory markers (Kruskal‐Wallis *p* = 0.055–0.87; Figure S2c). Nevertheless, thrombomodulin, C3dg, and C1s/C1‐INH complexes exhibited non‐significant trends toward higher levels with increasing histological severity, whereas MASP‐1/C1‐INH complexes tended to decrease.

Among patients with LN and available urine ACR measurements (*n* = 58), thrombomodulin showed a modest but statistically significant correlation with albuminuria (*ρ* = 0.29; *p* = 0.029; Figure S2d). In contrast, no association was observed between thrombomodulin and anti‐dsDNA levels within the LN subgroup (*ρ* = −0.02; *p* = 0.89; Figure S2e).

## 4 Discussion

In this large, well‐characterized multicenter cohort of patients with SLE, we identified endothelial activation as a central feature of the thrombo‐inflammatory profile associated with LN. Among the biomarkers examined, circulating thrombomodulin emerged as the most robust discriminator between SLE with and without renal involvement, remaining significantly elevated after adjustment for clinical confounders and correction for multiple testing and sensitivity analyses. This finding was consistent across univariate, multivariable, and network‐based analyses, underscoring its biological and statistical robustness. These findings identify circulating thrombomodulin as a robust marker associated with renal involvement and provide a rationale for longitudinal studies investigating its relationship with renal and thrombotic outcomes.

Thrombomodulin is a transmembrane glycoprotein expressed on endothelial cells and plays a key regulatory role in the protein C anticoagulant pathway [37, 38]. Elevated circulating thrombomodulin reflects endothelial injury and glycocalyx shedding and has been described in inflammatory, cardiovascular, and renal diseases [39]. Previous case reports have described improved renal outcomes after treatment with recombinant thrombomodulin in thrombotic microangiopathy associated with LN [40]. In patients with SLE, immune complex deposition, complement activation, and autoantibody‐mediated endothelial damage are recognized drivers of vascular dysfunction in LN [41]. Our findings extend previous work by demonstrating that thrombomodulin distinguishes LN from non‐renal SLE, even in a predominantly low‐disease‐activity cohort, and after adjustment for treatment exposure and APS status.

The modest association between thrombomodulin and albuminuria observed in patients with LN is consistent with experimental and clinical evidence linking proteinuria to endothelial glycocalyx disruption and vascular dysfunction [42]. The lack of association between global disease activity and thrombomodulin levels is consistent with previous reports suggesting that endothelial injury in LN may be partially uncoupled from systemic immunological activity [43, 44]. From a mechanistic perspective, shedding of endothelial thrombomodulin may contribute directly to a prothrombotic milieu by impairing activation of the protein C pathway and reducing endogenous anticoagulant capacity [37, 38]. However, the present study did not assess endothelial thrombomodulin expression or prospective thrombotic outcomes and therefore cannot determine whether elevated circulating thrombomodulin contributes causally to thrombosis in LN. Additionally, reduced protein C activity has previously been reported in SLE, partly attributed to aPL and anti–protein C antibodies [19]. Although we did not observe increased systemic thrombin generation in SLE‐LN after adjustment, likely influenced by antithrombotic therapy, localized or episodic hypercoagulability may still occur, as described in proteinuric renal disease [45].

To characterize global thrombo‐inflammatory interactions, we applied correlation network analysis, PCA, and multivariable logistic regression. Pairwise correlation networks revealed small interaction structures in SLE compared with none in disease control groups after FDR corrections. The observations may suggest coordinated interactions between endothelial injury, complement activation, coagulation, and fibrinolysis in SLE, rather than isolated pathway disturbances. However, most correlations were modest, and the findings could also represent differences in sample size; these network patterns should therefore be considered exploratory. In multivariable logistic regression analyses, thrombomodulin again showed the strongest association with LN. The stepwise model and the LASSO sensitivity analysis additionally retained markers related to lectin pathway activation, coagulation, and fibrinolysis, suggesting broader thrombo‐inflammatory involvement. Platelet activation markers were not retained in these models, suggesting that platelet activation does not primarily account for LN‐specific differences within SLE, despite its established role in SLE‐related thrombosis overall [19]. A similar pattern has been reported in nephrotic syndrome, where thrombomodulin, thrombin generation, and impaired fibrinolysis were markedly elevated in the absence of differences in platelet activation measures compared with controls [46]. Although we did not include functional assays on platelet activation and only three patients in our cohort had albuminuria in the nephrotic range (> 3500 mg/g), these parallels support the hypothesis that alterations in secondary haemostasis may be particularly relevant in LN and warrant further investigation.

Lectin‐pathway proteins did not differ significantly between SLE‐LN and SLE‐NonLN after correction for multiple testing. Although previous studies have suggested a potential role for CL‐K1 in kidney disease and LN [47, 48], we observed no consistent differences across kidney biopsy classes or between LN and non‐LN in our cohort. MASP‐1/C1‐INH complexes showed a non‐significant tendency to decrease with increasing histological severity, suggesting heterogeneity in complement pathway engagement across LN subtypes. However, these patterns were subtle and limited by small biopsy‐based sample sizes. Consistent with our findings, prior data from our group similarly demonstrated no association between MASP‐1/C1‐INH and kidney biopsy class in an independent Danish SLE cohort [49].

A key strength of the study is the inclusion of primary APS, primary SjD, and healthy individuals, which allowed the findings within SLE to be contextualized across a broader autoimmune spectrum. However, these comparator groups were not designed or powered for formal statistical testing, particularly given the small APS sample size. The observed platelet activation in APS aligns with experimental and clinical evidence implicating FcγRIIA‐mediated platelet activation in aPL‐driven thrombosis [19, 50]. Descriptively, SjD showed fewer nominal cross‐pathway interactions and biomarker levels generally closer to those of healthy controls. The more limited interaction patterns observed in SjD are consistent with its comparatively lower thrombotic risk [9, 10].

Our study has several limitations. First, its cross‐sectional design precludes causal inference and limits evaluation of temporal relationships. Residual confounding from treatment exposure and comorbidities cannot be excluded, as cumulative glucocorticoid dose and long‐term antithrombotic use were unavailable. Kidney biopsy data were also missing for a proportion of LN patients, introducing potential misclassification, although sensitivity analyses did not identify meaningful differences between groups. Second, the SLE cohort was pooled from two recruitment sites with differing inclusion periods, sample availability, and levels of clinical detail, introducing potential confounding related to clinical management, sample handling, and long‐term storage. Notably, the Odense cohort lacked citrated plasma and detailed prospective clinical data. However, the cohorts were broadly comparable, and sensitivity analyses excluding variables with unexplained between‐site differences did not materially change the results. Third, because the cohort was not originally designed for the present research purpose, information on important cardiovascular and lifestyle risk factors was unavailable, including smoking status, body mass index, and timing from LN onset to study inclusion. Also, thromboembolic outcomes were not systematically collected, preventing direct assessment of biomarker patterns in relation to future thrombotic events. Fourth, the relatively small sizes of the disease control groups and biopsy‐based subgroups reduced statistical power for certain comparisons and limited the interpretation of subgroup analyses. Differences in age between disease controls and the SLE cohort further restricted comparability. Fifth, the extensive biomarker panel and multiple analytical approaches increased the risk of Type I error, despite formal correction for multiple testing. Correlations were generally modest, and PCA captured only a limited proportion of variance; thus, these analyses should be viewed as exploratory and descriptive rather than evidence of causal mechanisms or predictive utility. Finally, the predominantly White European ancestry of the cohort may limit generalizability.

Despite these limitations, our findings suggest that circulating thrombomodulin represents a promising biomarker of endothelial injury in LN. Based on the present cross‐sectional data, we primarily consider circulating thrombomodulin a biomarker of endothelial injury associated with renal involvement in SLE. Its modest association with albuminuria raises the possibility that it may also reflect renal endothelial involvement, although this requires validation in future studies. Given the established role of endothelial thrombomodulin in coagulation regulation, the association between elevated circulating thrombomodulin levels and subsequent thrombotic risk warrants further investigation. Longitudinal studies are needed to determine whether thrombomodulin predicts renal flares, progressive kidney damage, or thrombotic events. Furthermore, the observed cross‐pathway interaction patterns support further investigation of therapeutic strategies targeting endothelial protection and coagulation‐complement crosstalk in LN.

## 5 Conclusion

LN is characterized by a distinct thrombo‐inflammatory profile with evidence of endothelial activation, and thrombomodulin emerging as the most consistent biological marker associated with renal involvement in SLE. These findings support further longitudinal and mechanistic studies of endothelial and thrombo‐inflammatory pathways in LN.

## Funding

This work was supported by Aarhus Universitet, 25051018. Gigtforeningen, R166‐A5554, R224‐A8313. A.P. Møller og Hustru Chastine Mc‐Kinney Møllers Fond til almene Formaal, L‐2023‐00094. Direktør Kurt Bønnelycke og Hustru fru Grethe Bønnelyckes Fond. Lundbeck Foundation, R264‐2017‐3344.

## Conflicts of Interest

None related to this manuscript. The authors report the following general conflicts of interest: M.L.L. reports receiving travel reimbursement from AstraZeneca; AV: PI in trials from BMS and UCB, received travel expenses from AstraZeneca; J.B.L. has received lecture honoraria (paid to her institution) from Bristol‐Myers Squibb and Merck and has received research funding to Aarhus University from Independent Research Fund Denmark and Karen Elise Jensens Fond; E.M.H. has received fees for speaking and/or consulting from Novo, Novartis, AbbVie, SynACT Pharma, UCB, research funding to Aarhus University Hospital from Independent Research Fund Denmark, Novo Nordisk Foundation, Danish Rheumatism Association, Aarhus University, Danish Regions Medicine Grants, Galapagos, AbbVie, SynACT Pharma, travel expenses from Sobi, AbbVie, Novartis, Boehringer‐Ingelheim, principal investigator in trials by SynACT Pharma and involved as site principal investigator in trials by AbbVie, Novartis, Novo Nordisk, Sanofi, AstraZeneca, Janssen‐Cilag; A.T. has collaborated with AstraZeneca on the PETUNIA study, has received honoraria for educational activities, and has served on advisory boards for AstraZeneca, Roche and Otsuka.

## Supporting information


**Table S1:** Characteristics of the two systemic lupus erythematosus recruitment sites; Aarhus University Hospital (Aarhus) and Odense University Hospital (Odense).
**Table S2:** Characteristics of patients with lupus nephritis (LN) according to availability of kidney biopsy data (Yes/No).
**Table S3:** Combined summary of the full and stepwise logistic regression models for lupus nephritis (LN) in the systemic lupus erythematosus (SLE) group. The full model includes all thrombo‐inflammatory variables and clinical covariates; the stepwise model retains variables providing the best model fit based on AIC, and the LASSO model based on penalized odds ratio (OR).
**Figure S1:** Spearman correlation matrices and correlation networks across systemic lupus erythematosus with lupus nephritis (SLE‐LN, a), SLE without nephritis (SLE‐nonLN, b), primary antiphospholipid syndrome (APS, c), and primary Sjögren's disease (SjD, d). Heatmaps show Spearman correlation coefficients (ρ), with unajusted significance indicated as *, **, *** for nominal *p* < 0.05, *p* < 0.01, and *p* < 0.001, respectively. Networks display only significant correlations (nominal *p* ≤ 0.05), with edge width proportional to |ρ| and red/blue edges denoting positive/negative associations. Biomarker nodes are grouped by biological subsystem.
**Figure S2:** (a) Principal component analysis (PCA) of the thrombo‐inflammatory panel across kidney biopsy WHO classes in patients with lupus nephritis (SLE‐LN) restricted to available WHO Class I–V kidney biopsy scores (complete‐case data). Points represent individual patients. PCA restricted to patients with both available biopsy classification and complete biomarker data (Class I–II, *n* = 3; Class III–IV, *n* = 30; Class V, *n* = 5). (b) PCA of the thrombo‐inflammatory panel across proteinuria, stratified by spot/day urine albumin/creatinine ratio (ACR) above or below 500 mg/g in SLE‐LN. Complete‐case data. Points represent individual patients. (c) Boxplots showing the distribution of selected thrombo‐inflammatory laboratory variables across WHO kidney biopsy classes in patients with LN. Boxes represent medians and interquartile ranges (IQR); whiskers represent 1.5 × IQR; points represent individual observations. Overall differences were assessed using Kruskal–Wallis tests. (d) Spearman correlation between thrombomodulin and spot urine Albumin/creatinine (A/C) ratio in SLE‐LN. (e) Spearman correlation between thrombomodulin and anti‐double stranded DNA antibody levels in SLE‐LN.
**Data S1:** STROBE Statement‐Checklist of items that should be included in reports of *cross‐sectional studies*.

## Data Availability

The data that support the findings of this study are available on request from the corresponding author. The data are not publicly available due to privacy or ethical restrictions.

## References

[1] A. Kaul , C. Gordon , M. K. Crow , et al., “Systemic Lupus Erythematosus,” Nature Reviews. Disease Primers 2 (2016): 16039. 10.1038/nrdp.2016.39 27306639

[2] S. R. Moe , H. Haukeland , C. Brunborg , et al., “Accrual of Thromboembolic Events and Antiphospholipid Syndrome in New‐Onset Systemic Lupus Erythematosus: A Population‐Based Inception Cohort Study,” RMD Open 11, no. 3 (2025): e005795. 10.1136/rmdopen-2025-005795 PMC12406804 40903053

[3] N. Bello , K. J. Meyers , J. Workman , J. Marcano Belisario , and R. Cervera , “Systematic Literature Review and Meta‐Analysis of Venous Thromboembolism Events in Systemic Lupus Erythematosus,” Rheumatology and Therapy 10, no. 1 (2023): 7–34. 10.1007/s40744-022-00513-1 PMC9931974 36471199

[4] L. Riancho‐Zarrabeitia , V. Martínez‐Taboada , I. Rúa‐Figueroa , et al., “Antiphospholipid Syndrome (APS) in Patients With Systemic Lupus Erythematosus (SLE) Implies a More Severe Disease With More Damage Accrual and Higher Mortality,” Lupus 29, no. 12 (2020): 1556–1565. 10.1177/0961203320950477 32807021

[5] M. Barbhaiya , S. Zuily , R. Naden , et al., “ACR/EULAR Antiphospholipid Syndrome Classification Criteria,” Arthritis and Rheumatology 82, no. 10 (2023): 1258–1270. 10.1136/ard-2023-224609 37640450

[6] M. G. Tektonidou , K. Laskari , D. B. Panagiotakos , and H. M. Moutsopoulos , “Risk Factors for Thrombosis and Primary Thrombosis Prevention in Patients With Systemic Lupus Erythematosus With or Without Antiphospholipid Antibodies,” Arthritis and Rheumatism 61, no. 1 (2009): 29–36. 10.1002/art.24232 19116963

[7] G. Ruacho , M. Kvarnström , A. Zickert , et al., “Sjögren Syndrome in Systemic Lupus Erythematosus: A Subset Characterized by a Systemic Inflammatory State,” Journal of Rheumatology 47, no. 6 (2020): 865–875. 10.3899/jrheum.190250 31523050

[8] C. H. Shiboski , S. C. Shiboski , R. Seror , et al., “2016 American College of Rheumatology/European League Against Rheumatism Classification Criteria for Primary Sjögren's Syndrome: A Consensus and Data‐Driven Methodology Involving Three International Patient Cohorts,” Arthritis & Rhematology 69, no. 1 (2017): 35–45. 10.1002/art.39859 PMC5650478 27785888

[9] I. Rúa‐Figueroa , M. Fernández Castro , J. L. Andreu , et al., “Comorbidities in Patients With Primary Sjögren's Syndrome and Systemic Lupus Erythematosus: A Comparative Registries‐Based Study,” Arthritis Care & Research (Hoboken) 69, no. 1 (2017): 38–45. 10.1002/acr.23015 27564390

[10] S. G. Pasoto , V. Adriano de Oliveira Martins , and E. Bonfa , “Sjögren's Syndrome and Systemic Lupus Erythematosus: Links and Risks,” Open Access Rheumatology and Research 11 (2019): 33–45. 10.2147/OARRR.S167783 PMC6357904 30774485

[11] J. G. Hanly , A. G. O'Keeffe , L. Su , et al., “The Frequency and Outcome of Lupus Nephritis: Results From an International Inception Cohort Study,” Rheumatology (Oxford) 55, no. 2 (2016): 252–262. 10.1093/rheumatology/kev311 PMC4939728 26342222

[12] R. Kaiser , C. M. Cleveland , and L. A. Criswell , “Risk and Protective Factors for Thrombosis in Systemic Lupus Erythematosus: Results From a Large, Multi‐Ethnic Cohort,” Annals of the Rheumatic Diseases 68, no. 2 (2009): 238–241. 10.1136/ard.2008.093013 PMC2875136 18782792

[13] I. Parodis , L. Arnaud , J. Gerhardsson , et al., “Antiphospholipid Antibodies in Lupus Nephritis,” PLoS One 11, no. 6 (2016): e0158076. 10.1371/journal.pone.0158076 PMC4919007 27336701

[14] S. E. Reppe Moe , Ø. Molberg , E. H. Strøm , and K. Lerang , “Assessing the Relative Impact of Lupus Nephritis on Mortality in a Population‐Based Systemic Lupus Erythematosus Cohort,” Lupus 28, no. 7 (2019): 818–825. 10.1177/0961203319847275 31072277

[15] K. Wattanakit and M. Cushman , “Chronic Kidney Disease and Venous Thromboembolism: Epidemiology and Mechanisms,” Current Opinion in Pulmonary Medicine 15, no. 5 (2009): 408–412. 10.1097/MCP.0b013e32832ee371 PMC2811494 19561505

[16] C. F. Christiansen , M. Schmidt , A. L. Lamberg , et al., “Kidney Disease and Risk of Venous Thromboembolism: A Nationwide Population–Based Case–Control Study,” Journal of Thrombosis and Haemostasis 12, no. 9 (2014): 1449–1454. 10.1111/jth.12652 25040558

[17] J. T. Gustafsson and E. Svenungsson , “Definitions of and Contributions to Cardiovascular Disease in Systemic Lupus Erythematosus,” Autoimmunity 47, no. 2 (2014): 67–76. 10.3109/08916934.2013.856005 24228980

[18] M. Bazzan , A. Vaccarino , and F. Marletto , “Systemic Lupus Erythematosus and Thrombosis,” Thrombosis Journal 13 (2015): 16. 10.1186/s12959-015-0043-3 PMC4407320 25908929

[19] M. L. Larsen , L. Nørgaard , P. Linge , et al., “Molecular Mechanisms Underlying Thrombosis in Systemic Lupus Erythematosus—A Systematic Review,” Seminars in Arthritis and Rheumatism 72 (2025): 152707. 10.1016/j.semarthrit.2025.152707 40086157

[20] D. Song , W. Y. Guo , F. M. Wang , et al., “Complement Alternative Pathway's Activation in Patients With Lupus Nephritis,” American Journal of the Medical Sciences 353, no. 3 (2017): 247–257. 10.1016/j.amjms.2017.01.005 28262211

[21] M. C. Hochberg , “Updating the American College of Rheumatology Revised Criteria for the Classification of Systemic Lupus Erythematosus,” Arthritis and Rheumatism 40, no. 9 (1997): 1725. 10.1002/art.1780400928 9324032

[22] A. Voss , A. Green , and P. Junker , “Systemic Lupus Erythematosus in Denmark: Clinical and Epidemiological Characterization of a County‐Based Cohort,” Scandinavian Journal of Rheumatology 27, no. 2 (1998): 98–105. 10.1080/030097498440958 9572634

[23] J. J. Weening , V. D. D'Agati , M. M. Schwartz , et al., “The Classification of Glomerulonephritis in Systemic Lupus Erythematosus Revisited,” Kidney International 15, no. 2 (2004): 241–250. 10.1097/01.asn.0000108969.21691.5d 14747370

[24] D. D. Gladman , D. Ibañez , and M. B. Urowitz , “Systemic Lupus Erythematosus Disease Activity Index 2000,” Journal of Rheumatology 29, no. 2 (2002): 288–291. 11838846

[25] D. D. Gladman , M. B. Urowitz , C. H. Goldsmith , et al., “The Reliability of the Systemic Lupus International Collaborating Clinics/American College of Rheumatology Damage Index in Patients With Systemic Lupus Erythematosus,” Arthritis and Rheumatism 40, no. 5 (1997): 809–813. 10.1002/art.1780400506 9153540

[26] S. Thiel , M. Møller‐Kristensen , L. Jensen , et al., “Assays for the Functional Activity of the Mannan‐Binding Lectin Pathway of Complement Activation,” Immunobiology 205, no. 4–5 (2002): 446–454. 10.1078/0171-2985-00145 12396006

[27] E. Axelgaard , L. Jensen , T. F. Dyrlund , et al., “Investigations on Collectin Liver 1,” Journal of Biological Chemistry 288, no. 32 (2013): 23407–23420. 10.1074/jbc.M113.492603 PMC3743509 23814060

[28] L. Selman , M. L. Henriksen , J. Brandt , et al., “An Enzyme‐Linked Immunosorbent Assay (ELISA) for Quantification of Human Collectin 11 (CL‐11, CL‐K1),” Journal of Immunological Methods 375, no. 1–2 (2012): 182–188. 10.1016/j.jim.2011.10.010 PMC3657160 22301270

[29] T. Wittenborn , S. Thiel , L. Jensen , H. J. Nielsen , and J. C. Jensenius , “Characteristics and Biological Variations of M‐Ficolin, a Pattern Recognition Molecule, in Plasma,” Journal of Innate Immunity 2, no. 2 (2010): 167–180. 10.1159/000218324 20375634

[30] A. Krarup , U. B. S. Sørensen , M. Matsushita , J. C. Jensenius , and S. Thiel , “Effect of Capsulation of Opportunistic Pathogenic Bacteria on Binding of the Pattern Recognition Molecules Mannan‐Binding Lectin, L‐Ficolin, and H‐Ficolin,” Infection and Immunity 73, no. 2 (2005): 1052–1060. 10.1128/IAI.73.2.1052-1060.2005 PMC547010 15664949

[31] A. Troldborg , A. Hansen , S. W. K. Hansen , J. C. Jensenius , K. Stengaard‐Pedersen , and S. Thiel , “Lectin Complement Pathway Proteins in Healthy Individuals,” Clinical and Experimental Immunology 188, no. 1 (2017): 138–147. 10.1111/cei.12909 PMC5343365 27925159

[32] S. E. Degn , L. Jensen , P. Gál , et al., “Biological Variations of MASP‐3 and MAp44, Two Splice Products of the MASP1 Gene Involved in Regulation of the Complement System,” Journal of Immunological Methods 361, no. 1 (2010): 37–50. 10.1016/j.jim.2010.07.006 20673767

[33] M. Møller‐Kristensen , J. C. Jensenius , L. Jensen , et al., “Levels of Mannan‐Binding Lectin‐Associated Serine Protease‐2 in Healthy Individuals,” Journal of Immunological Methods 282, no. 1–2 (2003): 159–167. 10.1016/j.jim.2003.08.012 14604549

[34] S. E. Degn , S. Thiel , O. Nielsen , A. G. Hansen , R. Steffensen , and J. C. Jensenius , “MAp19, the Alternative Splice Product of the MASP2 Gene,” Journal of Immunological Methods 373, no. 1–2 (2011): 89–101. 10.1016/j.jim.2011.08.006 PMC7099877 21871896

[35] A. Troldborg , L. Halkjær , H. Pedersen , et al., “Complement Activation in Human Autoimmune Diseases and Mouse Models; Employing a Sandwich Immunoassay Specific for C3dg,” Journal of Immunological Methods 486 (2020): 112866. 10.1016/j.jim.2020.112866 32941885

[36] A. Troldborg , Z. Godnic‐Polai , L. Cervenak , A. G. Hansen , H. Farkas , and S. Thiel , “Inter‐α‐Trypsin Inhibitor Heavy Chain 4 (ITIH4) as a Compensatory Protease Inhibitor in Hereditary Angioedema,” Journal of Allergy and Clinical Immunology 154, no. 2 (2024): 468–479. 10.1016/j.jaci.2024.03.028 38657796

[37] B. Dahlbäck and B. O. Villoutreix , “The Anticoagulant Protein C Pathway,” FEBS Letters 579, no. 15 (2005): 3310–3316. 10.1016/j.febslet.2005.03.001 15943976

[38] J. H. Griffin , B. V. Zlokovic , and L. O. Mosnier , “Protein C Anticoagulant and Cytoprotective Pathways,” International Journal of Hematology 95, no. 4 (2012): 333–345. 10.1007/s12185-012-1059-0 PMC3413316 22477541

[39] M. Boron , T. Hauzer‐Martin , J. Keil , and X. L. Sun , “Circulating Thrombomodulin: Release Mechanisms, Measurements, and Levels in Diseases and Medical Procedures,” TH Open 6, no. 3 (2022): e194–e212. 10.1055/a-1801-2055 PMC9273331 36046203

[40] K. Tonooka , H. Ito , T. Shibata , et al., “Recombinant Human Soluble Thrombomodulin for Treatment of Thrombotic Microangiopathy Associated With Lupus Nephritis,” Journal of Rheumatology 39, no. 8 (2012): 1766–1767. 10.3899/jrheum.111137 22859540

[41] S. Yung and T. M. Chan , “Endothelial Cell Activation and Glycocalyx Shedding ‐ Potential as Biomarkers in Patients With Lupus Nephritis,” Frontiers in Immunology 14 (2023): 1251876. 10.3389/fimmu.2023.1251876 PMC10579905 37854589

[42] A. H. J. Salmon , J. K. Ferguson , J. L. Burford , et al., “Loss of the Endothelial Glycocalyx Links Albuminuria and Vascular Dysfunction,” Journal of the American Society of Nephrology 23, no. 8 (2012): 1339–1350. 10.1681/ASN.2012010017 PMC3402289 22797190

[43] T. Witte , K. Hartung , C. Sachse , et al., “Thrombomodulin in Systemic Lupus Erythematosus: Association With Clinical and Laboratory Parameters,” Rheumatology International 19, no. 1–2 (1999): 15–18. 10.1007/s002960050092 10651075

[44] M. W. Boehme , M. W. J. Boehme , U. Raeth , P. R. Galle , W. Stremmel , and W. A. Scherbaum , “Serum Thrombomodulin‐a Reliable Marker of Disease Activity in Systemic Lupus Erythematosus (SLE): Advantage Over Established Serological Parameters to Indicate Disease Activity,” Clinical and Experimental Immunology 119, no. 1 (2000): 189–195. 10.1046/j.1365-2249.2000.01107.x PMC1905525 10606982

[45] K. I. Greenberg and M. J. Choi , “Understanding Hypercoagulability With Nephrotic Syndrome: How the Clot Thickens,” Clinical Journal of the American Society of Nephrology 18, no. 2 (2023): 149–151. 10.2215/CJN.0000000000000054 PMC10103297 36754000

[46] S. Kelddal , E. L. Grove , C. L. Duus , et al., “Alterations in Coagulation and Endothelial Function in Nephrotic Syndrome: A Multicenter, Cross‐Sectional Analysis,” Kidney360 6, no. 11 (2025): 1960–1969. 10.34067/KID.0000000865 PMC12626668 40478760

[47] A. Troldborg , S. Thiel , M. Trendelenburg , et al., “The Lectin Pathway of Complement Activation in Patients With Systemic Lupus Erythematosus,” Journal of Rheumatology 45, no. 8 (2018): 1136–1144. 10.3899/jrheum.171033 29907670

[48] S. Hansen , L. Selman , N. Palaniyar , et al., “Collectin 11 (CL‐11, CL‐K1) is a MASP‐1/3‐Associated Plasma Collectin With Microbial‐Binding Activity,” Journal of Immunology 185, no. 10 (2010): 6096–6104. 10.4049/jimmunol.1002185 20956340

[49] M. L. Larsen , A. Troldborg , E. J. M. Toonen , et al., “Differentiating Between Activation via the Lectin or the Classical Complement Pathway in Patients With Systemic Lupus Erythematosus,” Clinical and Experimental Immunology 214, no. 1 (2023): 18–25. 10.1093/cei/uxad070 PMC10711355 37407023

[50] I. Melki , I. Allaeys , N. Tessandier , et al., “FcgRIIA Expression Accelerates Nephritis and Increases Platelet Activation in Systemic Lupus Erythematosus,” Blood 136, no. 25 (2020): 2933–2945. 10.1182/blood.2020004974 PMC7751357 33331924

