# Supplementary material for: Thrombomodulin Shapes Thrombo‐Inflammatory Interactions in Lupus Nephritis
Source: Scand J Immunol. 2026 Oct 6;104(4):e70158. doi: 10.1111/sji.70158 (PMC13643033; doi:10.1111/sji.70158)
Supplement: Supplementary file 1 [file SJI-104-e70158-s001.docx]

**SUPPLEMENTARY MATERIAL**

**Table S1.** Characteristics of the two systemic lupus erythematosus recruitment sites; Aarhus University Hospital (Aarhus) and Odense University Hospital (Odense).

|  | **Variable** | **SLE (Aarhus)**  N = 203^1^ | **SLE (Odense)**  N = 122^1^ | **P-value**^2^ |
| --- | --- | --- | --- | --- |
| **General demographics** | Age | 47 (36, 59) | 47 (37, 57) | ns |
|  | Female sex | 184 (91%) | 109 (89%) | ns |
|  | Ethnicity, white | 191 (94%) | 115 (94%) | ns |
|  | Years of Disease | 10 (4, 21) | 10 (5, 17) | ns |
| **SLE characteristics** | ACR 1 (Malar Rash) | 139 (68%) | 66 (54%) | < 0.05 |
|  | ACR 2 (Discoid Lupus) | 20 (9.9%) | 4 (3.3%) | < 0.05 |
|  | ACR 3 (Photosensitivity) | 120 (59%) | 75 (61%) | ns |
|  | ACR 4 (Ulcers) | 92 (45%) | 28 (23%) | < 0.001 |
|  | ACR 5 (Arthritis) | 189 (93%) | 106 (87%) | ns |
|  | ACR 6 (Serositis) | 67 (33%) | 58 (48%) | < 0.05 |
|  | ACR 7 (Nephritis) | 60 (30%) | 50 (41%) | < 0.05 |
|  | Kidney biopsy available | 48 (24%) | NA |  |
|  | ACR 8 (CNS) | 19 (9.4%) | 20 (16%) | ns |
|  | ACR 9 (Hematological) | 171 (84%) | 74 (61%) | < 0.001 |
|  | ACR 10 (Immunological) | 190 (94%) | 107 (88%) | ns |
|  | Total ACR score | 6 (5, 7) | 6 (4, 7) | < 0.05 |
|  | SLEDAI score | 4 (2, 4) | 2 (0, 4) | < 0.001 |
|  | Physician global Assessment | 0 (0, 2) | NA |  |
|  | SLICC damage | 1(0, 2) | 1 (0, 3) | < 0.01 |
|  | Secondary APS | 32 (16%) | 39 (32%) | < 0.05 |
|  | Secondary SjD | 41 (20%) | NA |  |
| **Treatments at inclusion** | Prednisolone | 87 (43%) | 71 (62%) | < 0.05 |
|  | Hydroxychloroquine | 156 (77%) | 57 (61%) | < 0.05 |
|  | Antithrombotic Treatment | 85 (42%) | NA |  |
|  | Antiplatelet therapy | NA | NA |  |
|  | Anticoagulant therapy^3^ | NA | NA |  |
|  | Lipid-lowering therapy | 38 (19%) | NA |  |
|  | Anti-hypertensive therapy | 60 (30%) | NA |  |
| **Serology** | ANA positive | 202 (100%) | 122 (100%) | ns |
|  | Anti-dsDNA titer (IU/L) | 9 (2, 33) | NA |  |
|  | Lupus Anticoagulant positive | 56 (32%) | NA |  |
| **General laboratory values** | Lactate dehydrogenase (U/L) | 189 (164, 219) | NA |  |
|  | Alanine aminotransferase (U/L) | 21 (16, 28) | NA |  |
|  | creatinine (µmol/L) | 62 (55, 72) | NA |  |
|  | eGFR (mL/min/1.73 m²) | 90 (85, 90) | NA |  |
|  | C-reactive protein (mg/L) | 4.0 (4.0, 4.2) | NA |  |
|  | Immunoglobulin G (g/L) | 11.5 (9.4, 14.0) | NA |  |
|  | Leukocyte count (×10⁹/L) | 5.50 (4.40, 7.25) | NA |  |
|  | Lymphocyte count (×10⁹/L) | 1.27 (0.91, 1.74) | NA |  |
|  | Hemoglobin (mmol/L) | 8.40 (7.70, 8.80) | NA |  |
|  | Platelet count (×10⁹/L) | 237 (203, 280) | NA |  |
| **Complement analyses** | C3 (g/L) | 0.84 (0.70, 0.98) | 1.02 (0.84, 1.34) | < 0.001 |
|  | C4 (g/L) | 0.10 (0.06, 0.15) | 0.19 (0.11, 0.26) | < 0.001 |
|  | C3dg (mU/mL) | 59 (45, 81) | 43 (28, 58) | < 0.001 |
|  | MASP1/C1-INH complex (ng/mL) | 35 (25, 49) | NA |  |
|  | C1s/C1-INH complex (ng/mL) | 1244 (1005, 1568) | NA |  |
|  | MASP-3 (ng/mL) | 7029 (6220, 8327) | 5313 (4371, 6943) | < 0.001 |
|  | MASP-1 (ng/mL) | 12882 (10263, 15762) | 12392 (9950, 15275) | ns |
|  | H-ficolin (ng/mL) | 29698 (24050, 35596) | 23490 (19227, 30825) | < 0.001 |
|  | L-ficolin (ng/mL) | 2532 (2056, 3114) | 2742 (2382, 3362) | < 0.05 |
|  | MBL (ng/mL) | 1547 (619, 3030) | 1698 (590, 2899) | ns |
|  | M-ficolin (ng/mL) | 3639 (2725, 4877) | 3356 (2368, 4718) | ns |
|  | MASP-2 (ng/mL) | 311 (198, 456) | NA |  |
|  | MAp44 (ng/mL) | 3328 (2878, 3827) | 2949 (2637, 3542) | < 0.05 |
|  | Collectin-Liver-1 (ng/mL) | 541 (484, 601) | 529 (463, 572) | ns |
|  | Collectin-kidney-1 (ng/mL) | 349 (304, 402) | 320 (273, 373) | < 0.001 |
|  | IHIT-4 (µg/mL) | 231 (207, 261) | 213 (181, 239) | < 0.001 |
| **Coagulation analyses** | Prothrombin Fragment 1+2 (pmol/L) | 165 (108, 264) | NA |  |
|  | PAI-1 (ng/mL) | 3 (2, 7) | NA |  |
| **Endothelial analyses** | Syndecan-1 (ng/mL) | 62 (48, 80) | 71 (53, 99) | < 0.05 |
|  | Thrombomodulin (ng/mL) | 9.3 (7.3, 11.9) | 9.2 (6.6, 12.9) | ns |
| **Platelet analyses** | P-selectin (ng/mL) | 33 (25, 45) | 38 (30, 54) | < 0.05 |

^1^Median (Q1, Q3); n (%),^²^P-values computed using appropriate tests: Wilcoxon rank-sum, Welch t-test (on log-transformed data), or Fisher’s exact test depending on variable type, ^3^either vitamin K antagonist or direct oral anticoagulant treatment

APS: antiphospholipid syndrome, SLE: systemic lupus erythematosus, SjD: Sjögren’s disease, ANA: antinuclear antibody dsDNA: double-stranded DNA antibody, EULAR: European Alliance of Associations for Rheumatology, ACR: American College of Rheumatology, SLEDAI: Systemic Lupus Erythematosus Disease Activity Index, SLICC: Systemic Lupus International Collaborating Clinics Damage Index, C1-INH: C1-inhibitor, PAI-1: plasminogen-activator-inhibitor-1, ITIH-4: Inter-α-trypsin inhibitor heavy chain 4.

**Table S2.** Characteristics of patients with lupus nephritis (LN) according to availability of kidney biopsy data (Yes/No).

| **Characteristic** | **No**  N = 63^1^ | **Yes**  N = 47^1^ | **p-value**^2^ |
| --- | --- | --- | --- |
| Recruitment sites |  |  | <0.001 |
| Aarhus | 13 (21%) | 47 (100%) |  |
| Odense | 50 (79%) | 0 (0%) |  |
| Age | 45 (37, 61) | 40 (33, 48) | 0.03 |
| Female sex | 54 (86%) | 42 (89%) | Ns |
| SLEDAI Score | 2.0 (0.0, 4.0) | 4.0 (2.0, 6.0) | <0.001 |
| Years of Disease | 13 (7, 19) | 14 (6, 23) | Ns |
| Secondary APS | 20 (32%) | 6 (13%) | 0.02 |
| SLICC damage | 1 (1, 4) | 1 (0, 2) | 0.04 |
| Prednisone use | 42 (67%) | 28 (60%) | Ns |
| Spot-urine ACR (mg/g) | 50 (10, 97) | 104 (10, 541) | Ns |
| Urine ACR > 500 mg/g (spot/day) | 10 (16%) | 12 (26%) | Ns |
| ^1^Median (Q1, Q3); n (%) | | | |
| ^2^Wilcoxon rank sum test; Fisher's exact test | | | |

**Table S3**

Combined summary of the full and stepwise logistic regression models for lupus nephritis (LN) in the systemic lupus erythematosus (SLE) group. The full model includes all thrombo-inflammatory variables and clinical covariates; the stepwise model retains variables providing the best model fit based on AIC, and the LASSO model based on penalized odds ratio (OR).

| Predictor | Full model  OR (95% CI) | Full model p-value | Stepwise model  OR (95% CI) | Stepwise model p-value | LASSO model  (penalized OR) |
| --- | --- | --- | --- | --- | --- |
| Thrombomodulin (log) | 2.29 (1.13–4.63) | **0.021** | 2.35 (1.23–4.49) | **0.010** | 1.92 |
| Syndecan 1 (log) | 1.05 (0.56–1.96) | 0.891 |  |  |  |
| C3dg (log) | 1.14 (0.55–2.35) | 0.729 |  |  |  |
| MASP1/C1-INH complex (log) | 0.62 (0.34–1.13) | 0.121 | 0.66 (0.39–1.12) | 0.123 | 0.78 |
| C1s/C1-INH complex (log) | 0.92 (0.40–2.10) | 0.843 |  |  |  |
| MASP3 (log) | 1.23 (0.39–3.84) | 0.726 |  |  |  |
| Prothrombin Fragment 1+2 (log) | 0.70 (0.42–1.14) | 0.154 | 0.74 (0.51–1.07) | 0.107 | 0.84 |
| PAI-1 (log) | 1.15 (0.93–1.41) | 0.194 | 1.17 (0.95–1.43) | 0.141 | 1.08 |
| P-selectin (log) | 1.12 (0.64–1.97) | 0.696 |  |  |  |
| Age (years) | 0.97 (0.95–1.00) | 0.076 | 0.98 (0.95–1.00) | 0.066 | 0.98 |
| Anti-dsDNA (log) | 1.20 (1.01–1.41) | **0.033** | 1.19 (1.01–1.40) | **0.035** | 1.14 |
| Antithrombotic treatment (yes/no) | 1.49 (0.62–3.58) | 0.371 |  |  | 1.06 |
| Prednisone use (yes/no) | 2.14 (0.98–4.63) | 0.055 | 2.15 (1.02–4.51) | **0.044** | 1.85 |
| Secondary APS (yes/no) | 0.50 (0.14–1.85) | 0.302 |  |  |  |
| SLEDAI score | 1.12 (0.96–1.30) | 0.158 | 1.12 (0.98–1.28) | 0.110 | 1.09 |
| Akaike Information Criterion (model fit) | 223.0 | | 210.9 | | - |
| Area Under the Receiver Operating Characteristic Curve (AUC (ROC)) | 0.776 | | 0.773 | | 0.771 |

Confidence interval (CI), C1-inhibitor (C1-INH), plasminogen-activator inhibitor (PAI)-1, anti-double stranded DNA antibody titer value (anti-dsDNA), SLE disease activity index (SLEDAI)

**Figure S1 (a-d).** Spearman correlation matrices and correlation networks across systemic lupus erythematosus with lupus nephritis (SLE-LN, a), SLE without nephritis (SLE-nonLN, b), primary antiphospholipid syndrome (APS, c), and primary Sjögren's disease (SjD, d). Heatmaps show Spearman correlation coefficients (ρ), with unajusted significance indicated as *, **, *** for nominal p < 0.05, p < 0.01, and p < 0.001, respectively. Networks display only significant correlations (nominal p ≤ 0.05), with edge width proportional to |ρ| and red/blue edges denoting positive/negative associations. Biomarker nodes are grouped by biological subsystem.

a. **SLE-LN**

b. **SLE-NonLN**

c. **APS**

d. **SjD**
e.

**Figure S2 (a-e)**

a. Principal component analysis (PCA) of the thrombo-inflammatory panel across kidney biopsy WHO classes in patients with lupus nephritis (SLE-LN) restricted to available WHO class I–V kidney biopsy scores (complete-case data). Points represent individual patients. PCA restricted to patients with both available biopsy classification and complete biomarker data (class I–II, n=3; class III–IV, n=30; class V, n=5).
b. PCA of the thrombo-inflammatory panel across proteinuria, stratified by spot/day urine albumin/creatinine ratio (ACR) above or below 500mg/g in SLE-LN. Complete-case data. Points represent individual patients.
c. Boxplots showing the distribution of selected thrombo-inflammatory laboratory variables across WHO kidney biopsy classes in patients with LN. Boxes represent medians and interquartile ranges (IQR); whiskers represent 1.5×IQR; points represent individual observations. Overall differences were assessed using Kruskal–Wallis tests.
d. Spearman correlation between thrombomodulin and spot urine Albumin/creatinine (A/C) ratio in SLE-LN.
e. Spearman correlation between thrombomodulin and anti-double stranded DNA antibody levels in SLE-LN.

b.

e.

STROBE Statement-Checklist of items that should be included in reports of ***cross-sectional studies***

|  | Item No | Recommendation | Page No |
| --- | --- | --- | --- |
| **Title and abstract** | 1 | (*a*) Indicate the study’s design with a commonly used term in the title or the abstract | 2 |
|  |  | (*b*) Provide in the abstract an informative and balanced summary of what was done and what was found | 2 |
| Introduction | | | |
| Background/rationale | 2 | Explain the scientific background and rationale for the investigation being reported | 4 |
| Objectives | 3 | State specific objectives, including any prespecified hypotheses | 5 |
| Methods | | | |
| Study design | 4 | Present key elements of study design early in the paper | 5-8 |
| Setting | 5 | Describe the setting, locations, and relevant dates, including periods of recruitment, exposure, follow-up, and data collection | 5 |
| Participants | 6 | (*a*) Give the eligibility criteria, and the sources and methods of selection of participants | 5-6 |
| Variables | 7 | Clearly define all outcomes, exposures, predictors, potential confounders, and effect modifiers. Give diagnostic criteria, if applicable | 5 |
| Data sources/ measurement | 8* | For each variable of interest, give sources of data and details of methods of assessment (measurement). Describe comparability of assessment methods if there is more than one group | *5* |
| Bias | 9 | Describe any efforts to address potential sources of bias | 5-8 |
| Study size | 10 | Explain how the study size was arrived at | 5 |
| Quantitative variables | 11 | Explain how quantitative variables were handled in the analyses. If applicable, describe which groupings were chosen and why | 5-8 |
| Statistical methods | 12 | (*a*) Describe all statistical methods, including those used to control for confounding | 7-8 |
|  |  | (*b*) Describe any methods used to examine subgroups and interactions | 7-8 |
|  |  | (*c*) Explain how missing data were addressed | 7-8 |
|  |  | (*d*) If applicable, describe analytical methods taking account of sampling strategy | 7-8 |
|  |  | (*e*) Describe any sensitivity analyses | 7-8 |
| Results | | | |
| Participants | 13* | (a) Report numbers of individuals at each stage of study—eg numbers potentially eligible, examined for eligibility, confirmed eligible, included in the study, completing follow-up, and analysed | 8 |
|  |  | (b) Give reasons for non-participation at each stage | 8 |
|  |  | (c) Consider use of a flow diagram | - |
| Descriptive data | 14* | (a) Give characteristics of study participants (eg demographic, clinical, social) and information on exposures and potential confounders | 8-11  Tabl. 1 |
|  |  | (b) Indicate number of participants with missing data for each variable of interest | 8-11  Tabl. 1 |
| Outcome data | 15* | Report numbers of outcome events or summary measures | 8 |
| Main results | 16 | (*a*) Give unadjusted estimates and, if applicable, confounder-adjusted estimates and their precision (eg, 95% confidence interval). Make clear which confounders were adjusted for and why they were included | 8-11 |
|  |  | (*b*) Report category boundaries when continuous variables were categorized | 8-11 |
|  |  | (*c*) If relevant, consider translating estimates of relative risk into absolute risk for a meaningful time period | - |
| Other analyses | 17 | Report other analyses done—eg analyses of subgroups and interactions, and sensitivity analyses | 8-11 |
| Discussion | | | |
| Key results | 18 | Summarise key results with reference to study objectives | 11 |
| Limitations | 19 | Discuss limitations of the study, taking into account sources of potential bias or imprecision. Discuss both direction and magnitude of any potential bias | 12 |
| Interpretation | 20 | Give a cautious overall interpretation of results considering objectives, limitations, multiplicity of analyses, results from similar studies, and other relevant evidence | 13 |
| Generalisability | 21 | Discuss the generalisability (external validity) of the study results | 12-13 |
| Other information | | | |
| Funding | 22 | Give the source of funding and the role of the funders for the present study and, if applicable, for the original study on which the present article is based | 14-15 |
